# Extensive Spinal Cord Infarction: A Rare Challenging Entity

**DOI:** 10.31662/jmaj.2023-0168

**Published:** 2023-12-11

**Authors:** Vitorino Modesto dos Santos, Taciana Arruda Modesto Sugai

**Affiliations:** 1 Armed Forces Hospital, and Catholic University of Brasília-DF, Brazil; 2 Specialist, American Society of Neurophysiology, and Dermatologist of Brasília-DF, Brazil

**Keywords:** extensive myelopathy, longitudinal lesion, spinal cord infarction

Dear Editor,

We read an illustrative case study of a spinal cord infarction (SCI) with a length of 13 vertebral bodies in a 78-year-old female, published in this journal by Yashita D, et al. ^(1)^ Because the mean measure of SCIs is approximately three vertebral bodies, the authors hypothesized that the development of an arteriosclerotic occlusion at the great posterior radiculomedullary artery may have caused the development of an exceeding long infarction ^(1)^.

Our aim is to comment on recent literature about challenging extensive SCIs ^(2), (3), (4), (5)^. Chiew YR, et al. reported the case study of a 58-year-old male, who had the diagnosis of an extensive SCI established by the contrast-enhanced thoracic spine MRI diffusion-weighted imaging and apparent diffusion coefficient (DWI and ADC) evaluations ^(2)^. The authors emphasized the differences between longitudinally extensive lesions of SCI and neuromyelitis optica (NMO); the lack of contrast enhancement in SCI is due to disrupted blood flow, and in NMO it occurs by inflammation and the intact blood flow ^(2)^. Costamagna G, et al. described a 57-year-old female with longitudinally extensive transverse myelopathy by SCI, but a negative MRI DWI and ADC at 9 h after onset ^(3)^. As the manifestations persisted, the exam repeated 72h later revealed a hyperintense “pencil-like” signal mainly from T1-T6 on T2 sequence, and with restricted diffusion ^(3)^. The authors stressed the clinical indicative data of potential SCI overriding the initially negative MRI findings and should not cause delay timely and appropriate management ^(3)^. Naik A, et al. reviewed data of SCIs among 147 patients, 55.4% men, and mean age of 45 years; 47.3% of cases were idiopathic, and 19.6% were due to vascular pathology ^(4)^. The authors concluded that the initial MRI images may be normal, the SCIs between T4 and T7 and those due to trauma or systemic/chronic causes have worst outcomes, and the cerebrospinal fluid drainage and antiplatelet aggregate therapy are more effective tools ^(4)^. Stenimahitis V, et al. evaluated data of 57 patients aged 18 years or older and presenting SCIs between 2006 and 2019; 30 had spontaneous SCIs, and 27 periprocedural SCIs ^(5)^. They highlighted that MRI DWI is the main tool for diagnosis; spontaneous SCIs often affect a unique segment and the periprocedural are more extensive, with more impairment and longer hospital stay; and the main goal of management is preventing complications ^(5)^.

Concluding, the authors believe that the comments here emphasize the cornerstone practical points well described by the authors of the initial reference.

## Article Information

### Conflicts of Interest

None

### Author Contributions

VMS wrote the first manuscript, and TAMS edited and approved the final manuscript.

### Approval by Institutional Review Board (IRB)

IRB approval was not required for this manuscript.

### Informed Consent

Informed consent was not required for this study.
